# Effect of VIPS fabrication parameters on the removal of acetic acid by supported liquid membrane using a PES–graphene membrane support

**DOI:** 10.1039/c8ra03392g

**Published:** 2018-07-16

**Authors:** Norlisa Harruddin, Syed M. Saufi, Che Ku M. Faizal, Abdul Wahab Mohammad

**Affiliations:** Faculty of Chemical and Natural Resources Engineering, Universiti Malaysia Pahang Lebuhraya Tun Razak, 26300 Gambang Pahang Malaysia smsaufi@ump.edu.my; Faculty of Engineering Technology, Universiti Malaysia Pahang Lebuhraya Tun Razak, 26300 Gambang Pahang Malaysia; Department of Chemical and Process Engineering, Faculty of Engineering and Built Environment, Universiti Kebangsaan Malaysia 43600 Bangi Selangor Darul Ehsan Malaysia

## Abstract

In this study, the removal of acetic acid by supported liquid membrane (SLM) using hybrid polyethersulfone (PES)–graphene membrane prepared by vapor induced phase separation (VIPS) was investigated. The effects of graphene loading, coagulation bath temperature, air exposure time, and air humidity on the morphology, mechanical strength, porosity, and contact angle of the membrane were analyzed. The performance and stability of the hybrid membrane as a SLM support for acetic acid removal were studied. The best PES–graphene membrane support was produced at a coagulation bath temperature of 50 °C, an air exposure time of 30 s and air humidity of 80%. The fabricated membrane has a symmetrical micropore cellular structure, high porosity and high contact angle. Under specific SLM conditions, almost 95% of acetic acid was successfully removed from 10 g L^−1^ aqueous acetic acid solution. The hybrid membrane remains stable for more than 116 h without suffering any membrane breakage during the continuous SLM process.

## Abstract 2

In this study, the removal of acetic acid by supported liquid membrane (SLM) using hybrid polyethersulfone (PES)–graphene membrane prepared by vapor induced phase separation (VIPS) was investigated.

## Graphical abstract

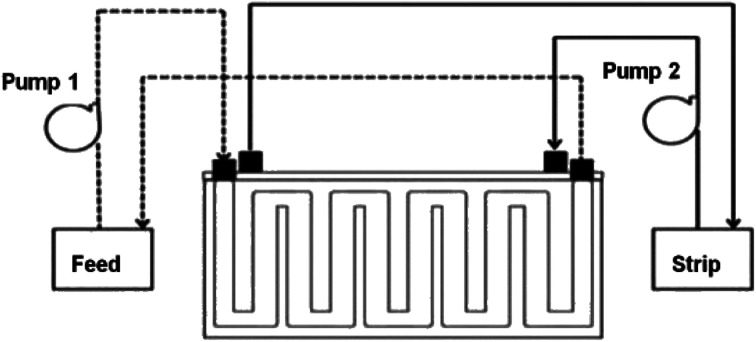

## 1 Introduction

Lignocellulosic biomass is an abundant organic material that can be used for sustainable production of biofuels, bioenergy and value added fine chemicals.^1,2^ Lignocellulosic biomass has to be hydrolyzed into fermented sugars before converting them to high value products through the fermentation process. The most common methods used to hydrolyze lignocellulosic biomass are by using acid hydrolysis. Sulphuric, hydrochloric or phosphoric acid are typically used in the hydrolysis at a moderate temperature around 100–150 °C and at an acid concentration of 1–10%.^3^ However, other byproducts such as furfural, hydroxymethylfurfural and acetic acids are also produced along with the sugars during the biomass hydrolysis. The formation of these compounds can inhibit the microorganism used in the fermentation process later. Acetic acid (AA) is found in large amounts in lignocellulosic biomass hydrolysate and considered as a serious inhibitor.^4^ Therefore, it must be removed from the biomass hydrolysate before the fermentation process.

Various methods including nanofiltration,^3,5^ reverse osmosis,^5,6^ and reactive extraction^7,8^ have been proposed to remove acetic acid. However, each of these methods has their own restrictions such as high operating pressure, membrane fouling, low selectivity, and high energy consumption.^9^ The SLM process shows a great potential in removal and recovery of a desired solute. The removal and recovery processes in SLM occur in one single step, thus providing maximum driving force for the separation of desired solutes with high recovery rates. Nowadays, very few researchers had focused on the removal of AA from an aqueous phase using the SLM process.^10^

In SLM process, the polymeric membrane support plays an important role in the transport and performance of the process. For an efficient immersion of the organic liquid membrane phase inside the support, the microporous polymeric membrane with small pore size, high porosity, high tensile strength, high hydrophobicity and highly resistant to chemical should be used.^11,12^ Membrane support with high hydrophobicity is required to retain and keep an organic liquid membrane phase stable within the membrane pores by capillary action force.^11^ Hence, a development of microporous membrane support with suitable characteristic is critical in order to achieve excellent separation efficiency using SLM process. Previous studies had showed that incorporation of graphene into polymer matrix can enhance the hydrophobicity, chemical stability and tensile strength of the membrane.^13–15^ Therefore, graphene was selected as a filler to prepare hybrid PES membrane support in the current study to remove AA using SLM process.

The most common method to fabricate microporous membrane support is through phase inversion process. VIPS is one of the approaches that is used in the phase inversion process. In VIPS process, the membrane gel is exposed to a humid air at certain period before immersion into a coagulation bath. Phase separation occurs when the water associated with the humid air transfer to the membrane gel film.^16^ VIPS is widely applied in membrane manufacturing and offers several advantages such simplicity, low cost method and highly efficient technique to produce porous membrane.^17^ Chen *et al.*^18^ had produced microporous PES hollow fiber membrane with sponge like structure by using VIPS technique for filtering bovine serum albumin (BSA). Adjusting air humidity, air gap distance and CBT during VIPS process had significantly affected the permeation flux and BSA rejection. Hence, this method is suitable for the fabrication of membrane support for SLM application.

To date, most studies on the removal of acetic acid using SLM process were based on the commercial membrane support. Therefore, this study focused on the removal of acetic acid using a custom-made membrane prepared through VIPS process. In order to increase the membrane hydrophobicity, graphene nanofiller was blend into the membrane solution to make hybrid PES–graphene membrane support. The incorporation of graphene in polymer solution was challenged because graphene sheets are difficult to disperse due to strong van Der Waals between the fillers.^19^ To the best of our knowledge, no work has been reported on fabrication PES membrane incorporation with graphene using VIPS technique. The main parameters influenced the morphology and physical characteristic of the membrane such as concentration of graphene, coagulation bath temperature (CBT), exposure time and air humidity were studied.

## 2 Experimental procedure

### 2.1 Materials

PES (Radel A300) supplied by Amoco Chemicals, was used as a membrane material. The polymer was dried for 24 h at 60 °C. Dimethylacetamide (DMAc) purchased from Merck (Darmstadt, Germany) and polyethylene glycol (PEG 200) purchased from Sigma (St. Louis, MO) were used as solvent and nonsolvent, respectively, in dope polymer solution. Tap water was used as a coagulation medium in VIPS process. Meanwhile, graphene nanopowder was used as an inorganic filler in dope polymer solution where it was kindly supplied by the Low Dimensional Materials Research Centre, Universiti Malaya, Malaysia. In organic liquid membrane phase, tri-*n*-octylamine (TOA) and 2-ethyl-1-hexanol were used as a carrier and diluent, respectively. Both chemicals were supplied by Sigma Aldrich. Acetic acid and sodium hydroxide were used in the feed phase and as a stripping agent, respectively, in the SLM experiment. Both chemicals were obtained from Merck (Darmstadt, Germany).

### 2.2 Membrane fabrication

The hybrid PES/graphene membrane was prepared by VIPS method. At first, the graphene nanoparticles were dispersed in DMAc by sonicating the mixture for 1 h. Sonication process can create shear stress and cavitation of graphene in the solvent. Then, under continuous stirring condition, PEG 200 and PES pellets were added to the mixture. This mixture was stirred for 48 h at room temperature to obtain a homogenous dope solution. Finally, the homogenous casting solution was degassed by putting it into the ultrasonic water bath for 24 h. The composition of the base polymer solution was 15 wt% of PES, 42.5% of DMAc and 42.5% of PEG 200. Different concentration of graphene from 0.1 to 1.0 wt% relative to the weight of PES were added into dope solution.

The dope solution was cast onto a glass plate to form a membrane gel film with 380 μm thickness using semi-automatic casting machine. The membrane film was then exposed in the controlled air environment with relative humidity (RH) in the range of 70 to 100%. The exposure time in the humid air varied between 10 and 70 s. Then, the cast film was immersed into water coagulation bath at different temperatures, from 30 to 60 °C to induce solidification process. The membrane was finally dried at room temperature for 48 h.

### 2.3 Characterization of membrane

#### 2.3.1 Membrane morphology

Scanning electron microscopy (SEM) (Brand: Carl Zeiss, Model: EVO 50) was used to analyze the cross sectional and morphology of the membrane. For the membrane prepared at different graphene concentration, field emission scanning electron microscope (FESEM) (Brand: JEOL, Model JSM 7800F) was used. The membrane samples were fractured in liquid nitrogen and sputtered with gold before visualizing under the SEM or FESEM machine.

#### 2.3.2 Contact angle

The hydrophilicity of the membrane was measured using CAM 101 Optical Contact Angle Meter, KSV Instruments. Membrane sample was placed on the top of glass slide and a droplet of 5 μL of ultrapure water was carefully deposited to membrane surface using ‘I’ shaped needle. A static image of the water droplet on the membrane surface was captured and analyzed using image analysis software to calculate the contact angle value. The measurement was performed at three different locations, and then, an average value was reported.

#### 2.3.3 Porosity

The membrane with dimension of 10.5 cm × 4 cm was dried in a vacuum oven at 80 °C for 24 h to remove all the water presence in the membrane pores. The dried membrane was weighted as *W*_1_. Dried membrane was then immersed in olive oil for 24 h. The excess oil on the wet surface of the membrane was absorbed using filter paper and the wet membrane was weighted as *W*_2_. The membrane overall porosity was calculated using eqn (1).1
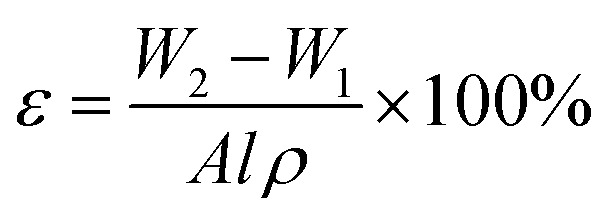
where, *W*_2_, is the weight of the wet membrane (kg); *W*_1_, is the weight of the dry membrane (kg); *A*, is the effective area of membrane (m^2^); *l*, is the thickness of membrane (m); *ρ*, is the density of olive oil (kg m^−3^).

#### 2.3.4 Mechanical strength

Mechanical properties of the membranes were measured by universal testing machine (INSTRON NVLAP) at a loading velocity of 5 mm min^−1^. The dry membrane samples were prepared in a rectangular shape with a membrane length of 50 mm and a width of 20 mm. The data of load (N)–extension (mm) of the membrane were obtained.

### 2.4 Supported liquid membrane process

#### 2.4.1 Preparation of solution

SLM consists of three phases, which are feed phase, organic liquid membrane phase and stripping phase. An aqueous AA of 10 g L^−1^ was used in the feed phase. The organic liquid membrane phase was composed of 0.1 M TOA carrier in 2-ethyl-1-hexanol diluent. For stripping phase, an aqueous 0.1 M sodium hydroxide was used.

#### 2.4.2 SLM process

**Fig. 1:**
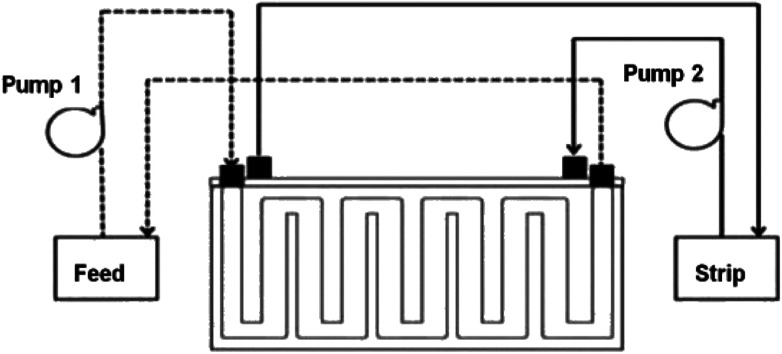
Schematic diagram of SLM system configuration.

The PES–graphene membrane support was impregnated in the organic liquid membrane for 24 h. The impregnated membrane was placed and clamped between two parts of the membrane cell as shown in Fig. 1. The inserted membrane acted as the boundary that separated the feed and stripping side in the SLM system. The feed and strip phase, 150 mL each, were circulated through the membrane cell at 50 mL min^−1^. Every hour, 1.5 mL of AA sample was taken from feed phase for analysis using high performance liquid chromatography analysis. The acetic acid removal percentage was calculated using eqn (2).2
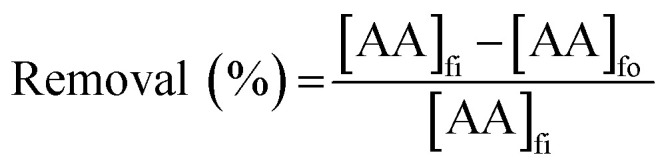
where, [AA]_fi_ and [AA]_fo_, represent the initial and the final concentration of AA (g L^−1^) in the feed phase, respectively.

#### 2.4.3 Membrane stability evaluation

The stability of the membrane was determined by running the SLM experiment continuously up to 112 h without reimpregnation the membrane support with the new organic liquid membrane. The feed and strip phases were renewed every 6 h and AA sample was taken every 2 h. The concentration of AA was determined by using high performance liquid chromatography analysis.

#### 2.4.4 High performance liquid chromatography analysis

The concentration of AA was detected by Synergy Hydro C18 HPLC column (150 mm × 4.6 mm × 4 μm) connected to Waters Acquity Ultra Performance Liquid Chromatography (UPLC) system. 0.02 M potassium dihydrogen phosphate was used as mobile phase and AA was detected by UV detector at 221 nm wavelength.

## 3 Results and discussion

### 3.1 Incorporation of graphene in the membrane support

Graphene was selected as an inorganic filler in this study to improve the morphology, hydrophobicity and mechanical strength of the membrane support for SLM process. Different concentrations of graphene from 0.1 to 1.0 wt% were blended into dope solution to find the best graphene loading. The membranes were casted at CBT of 40 °C, air exposure time of 30 s and air humidity of 80%.

#### 3.1.1 Membrane structure

**Fig. 2:**
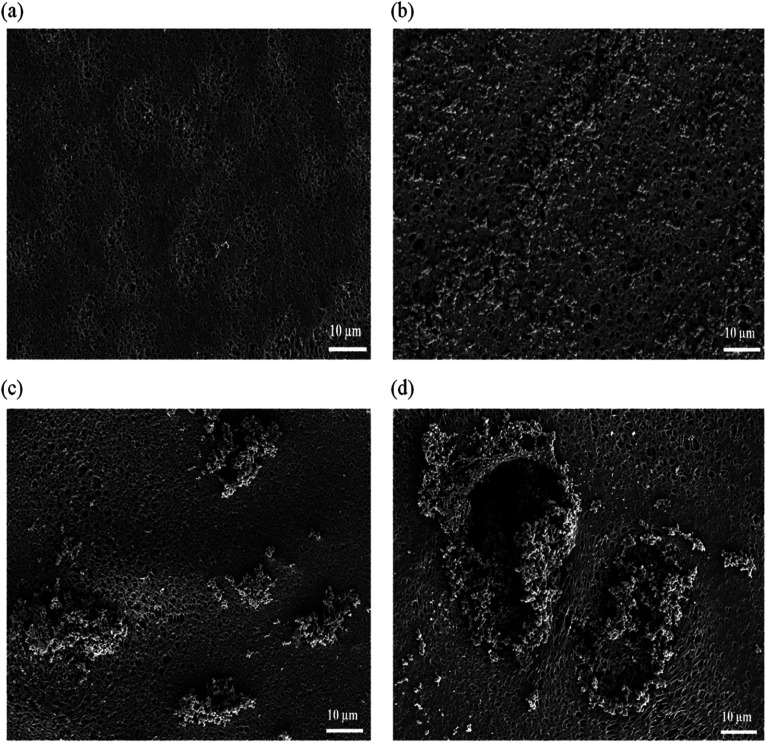
FESEM images of top surface membrane, (a) pristine PES, (b) PES/0.1 wt% graphene, (c) PES/0.5 wt% graphene, (d) PES/1.0 wt% graphene.

Fig. 2 exhibit top surface of the pristine PES membrane and hybrid PES–graphene membrane loaded with 0.1, 0.5 and 1.0 wt% graphene. Pristine PES membrane has a smooth top surface with scattered open pores as shown in Fig. 2(a). The surface roughness of the membrane was altered when the graphene was added into the membrane. At low loading of 0.1 wt%, surface roughness was increased but the graphene particles are well distributed on the membrane surface as shown in Fig. 2(b). Similar result was obtained by Dizaji *et al.*,^20^ who found that the graphene uniformly dispersed on the surface of polydimethylsiloxane (PDMS) matrix at low concentration of graphene. Well dispersed of graphene on the membrane surface can substantially improve the mechanical and physical properties of the hybrid membranes.^21^

However, further increase on the graphene content to 0.5 and 1.0 wt% resulted in small agglomeration of graphene at the membrane surface as shown in Fig. 2(c) and (d). This phenomenon occur due to van der Walls force among the neighboring particles of graphene.^22,23^ In previous study,^20^ graphene agglomeration occurred on the surface of PDMS–graphene/PES membrane when the concentration of filler increased up to 0.6 wt%. Agglomeration and random dispersion of the graphene on the membrane surface can interrupt the ability of graphene to exert its full potential in the membrane performance.

**Fig. 3:**
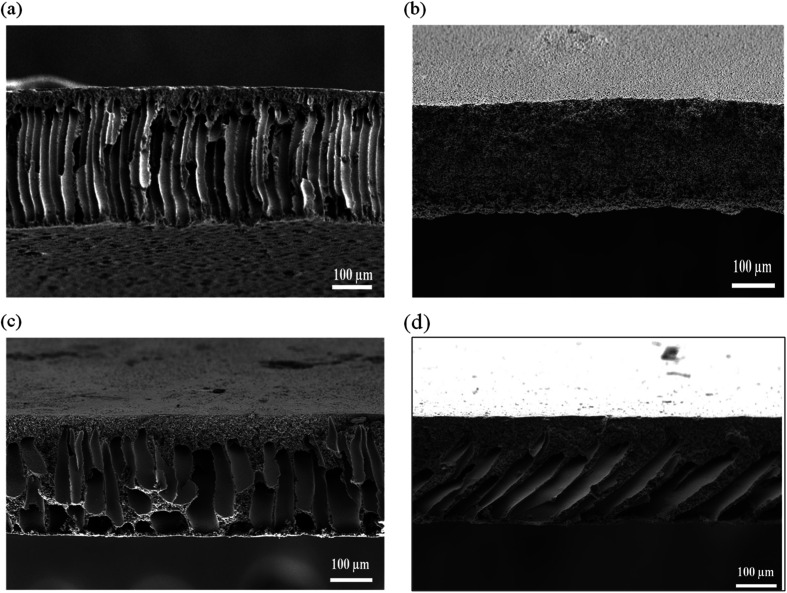
FESEM images of cross sectional membrane, (a) pristine PES, (b) PES/0.1 wt% graphene, (c) PES/0.5 wt% graphene, (d) PES/1.0 wt% graphene.

The cross sectional of the pristine PES membrane and PES–graphene membrane was showed in Fig. 3. Pristine PES membrane had an asymmetric structure composed of spongy like pores near the top skin followed by long cylindrical microvoids that uniformly distributed throughout the cross section of the membrane. Apparently, addition of low content of graphene filler (0.1 wt%) had significantly changed the overall membrane structure into a symmetric structure with a bicontinuous micropores (Fig. 3(b)). This pore structure is favorable as a support for the SLM system. However, the increment of the graphene loading to 0.5 and 1.0 wt% had induced the formation of spongy top skin layer and finger like macrovoids at the bottom layer of membranes as shown in Fig. 3(c) and (d).

#### 3.1.2 Membrane contact angle

Contact angle value for the pristine PES and hybrid PES–graphene membrane was showed in Table 1. The average contact angle of pristine PES membrane is 81.92°, which considered as hydrophilic membrane. The incorporation of the graphene was found significantly improved the hydrophobicity of the hybrid membrane. Adding 0.1 wt% of graphene produced highly hydrophobic hybrid membrane with contact angle value of 122.35°. Graphene is a single atom-thick sheet composed of sp^2^ hybridized carbon atom which is highly hydrophobic material.^24^ Hence, the incorporation of this filler can increases the contact angle value of the membrane. In addition, the surface roughness of the membrane was increased due to the existence of the graphene filler which eventually contributed to the increment of the membrane hydrophobicity.^15,25^ Furthermore, well dispersion of graphene on the membrane surface at low loading of 0.1 wt% as shown in Fig. 2(b) allows the graphene to function effectively. However, further increment of the graphene content up to 1.0 wt% had decreased the contact angle value to 100.92°. This decrement is due to the agglomeration of graphene on the membrane matrix as shown in Fig. 2(c and d). Graphene agglomeration might hindered graphene reactivity and ability to improve the membrane hydrophobicity.^23^

**Table 1:** Contact angle (°) value of the pristine PES membrane and hybrid PES–graphene membrane

Membrane support	Contact angle (°)
Pristine PES	81.92 ± 1.22
PES/0.1 wt% graphene	122.35 ± 2.14
PES/0.5 wt% graphene	108.61 ± 7.38
PES/1.0 wt% graphene	100.92 ± 0.37

#### 3.1.3 Mechanical strength

**Fig. 4:**
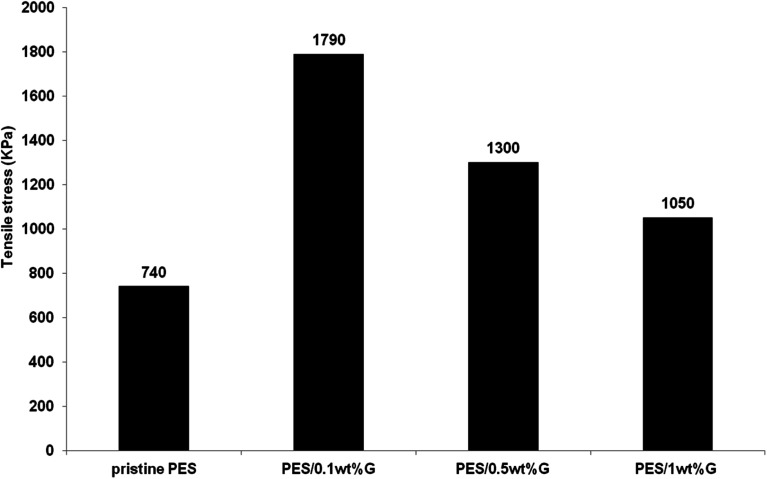
Maximum tensile stress (kPa) of pristine PES membrane and hybrid PES–graphene membrane.

Fig. 4 exhibits the tensile stress of PES membrane and hybrid PES–graphene membranes. The pristine PES membrane shows a lowest tensile stress of 740 kPa. Incorporation of graphene into the PES membrane had improved the mechanical strength of hybrid membranes dramatically. The best graphene loading was found at 0.1 wt% which gives the tensile stress of 1790 kPa, an improvement about 140% compared to pristine PES membrane. However further increases of the graphene content to 1 wt% had decreased the tensile stress to 1050 kPa. Papageorgiou *et al.*,^26^ found that mechanical strength is heavily affected by filler agglomeration especially at high filler concentration. Although the strength value was declined as the graphene loading increased, but it still higher compared to the pristine PES membrane. Based on the above discussion, hybrid PES–graphene membrane with 0.1 wt% loading was selected as the best dope formulation for further study on the membrane fabrication parameters.

### 3.2 Membrane morphological at different VIPS parameters

#### 3.2.1 Effect of coagulation bath temperature

**Fig. 5:**
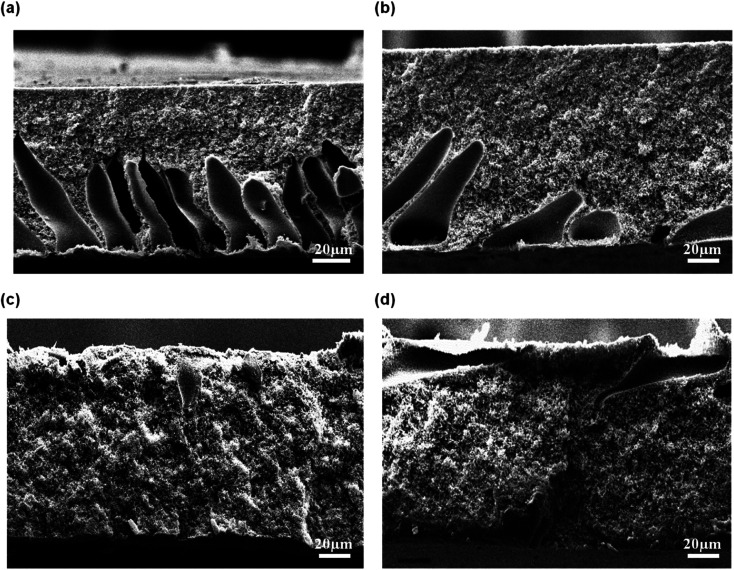
Cross-sectional of flat sheet membrane prepared at different CBT: (a) 30 °C, (b) 40 °C, (c) 50 °C, and (d) 60 °C.

**Fig. 6:**
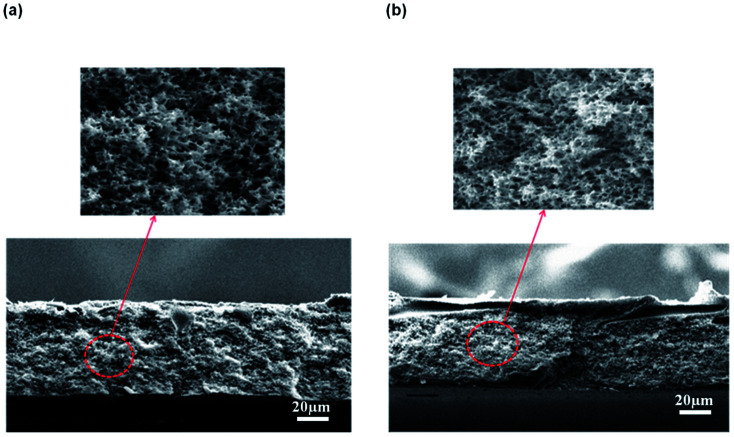
Micropores structure of the membrane support prepared at a CBT: (a) 50 °C and (b) 60 °C at 3000× magnification.

Fig. 5 shows the morphology of the membrane prepared at different CBT visualized by SEM at 300 × magnification. The air exposure time and relative humidity were kept constant at 30 s and 80%, respectively. Briefly, the macrovoids progressively disappeared with an increment of CBT from 30 °C to 60 °C. When the membranes were immersed at CBT of 30 °C, many large sized macrovoids were formed from middle to bottom part of the membrane. As the CBT increased to 40 °C, only few macrovoids were formed. Further increase of CBT to 50 °C and 60 °C completely suppressed the macrovoids and formed a bicontinuous morphology with well-connected pores. Similar trends were obtained by Xu *et al.*^27^ and Curcio *et al.*^28^ that showed an increasing of CBT induced transition of macrovoids finger-like structure into bicontinuous structure in their membrane. This bicontinuous structure can be seen more clearly at SEM image enlarged at 3000× magnification as shown in Fig. 6(a) and (b) for the membrane immersed at CBT of 50 °C and 60 °C, respectively.

The formation of the macrovoids and microporous structure can be explained by the phase inversion of the kinetics theory. Immersion process of the membrane solution into the coagulation bath is a demixing process. The demixing process occurs when the nuclei of polymer lean phase continue to grow with the continuation of the non-solvent and solvent exchange until the polymer concentration reaches a high level and solidification occurs. At this stage, the demixing process is completed.^27^ The level of the demixing process affects the membrane structures significantly. Low CBT causes instantaneous demixing process, which leads to the formation of macrovoids in the membrane structure, as shown in Fig. 5(a and b). Meanwhile, high CBT can delay the demixing process after a certain period of time, to which it can lead to the bicontinuous cellular structure formation.^29^ At this stage, large number of nuclei are created and distributed throughout the membrane cross-section and meanwhile, free growths of nuclei on the bottom layer are prevented.^7^ Besides, it can also enhance the micropores formation on membrane surface. This structure can be considered as symmetric membrane structure since it has uniform pore structure throughout the membrane cross-section.

#### 3.2.2 Effect of exposure time

**Fig. 7:**
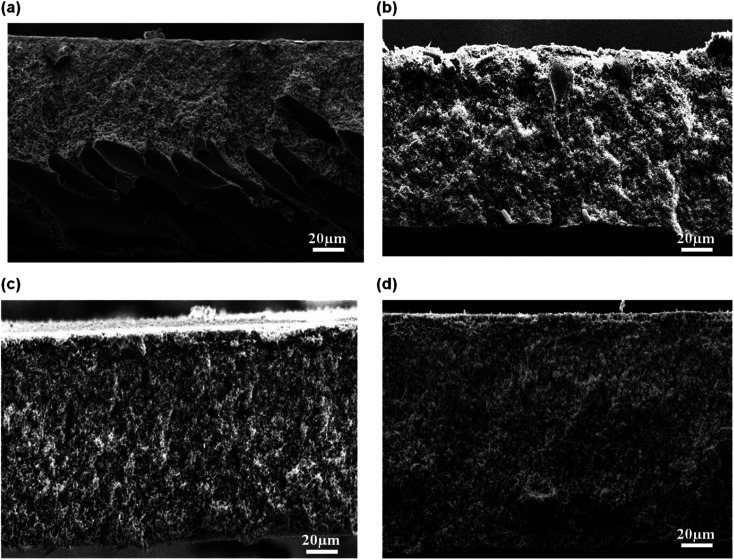
Cross-sectional of flat sheet membrane prepared at different exposure times: (a) 10 s, (b) 30 s, (c) 50 s, and (d) 70 s.

An exposure time is often introduced on the cast film before immersion into the coagulation bath. In this study, the cast films were exposed at 80% air relative humidity over a certain exposure time between 10 s to 70 s and later, immersed into water coagulation bath at 50 °C. The resultant morphology of the membranes is shown in Fig. 7. Based on Fig. 7(a), asymmetric membrane structure consists of large finger-like macrovoids and microporous sublayer was formed at 10 s air exposure time. At this moment, the absorbed water from the surrounding was insufficient to cause phase separation in the entire film. When the film was immersed into the water bath, cellular and digitate microvoids are formed. When the exposure time was lengthened to more than 30 s, the large macrovoids completely disappeared and a bicontinuous cellular membrane structure was formed, as visualized in Fig. 7(c) and (d). Adequate water absorption occurred at evaporation time above 30 s and that had caused the crystallization phase separation in the entire membrane film. In fact, it can be observed that during the casting process, the film turned to cloudy white when the exposure time was longer than 30 s.

At short exposure time, a delayed liquid–liquid demixing dominated. Hence, the formation of nuclei had occurred in a short time before the immersion into coagulation bath, thus leading to the development of open cell macrovoids. Liquid–liquid demixing had accelerated as the exposure time was increased and that had caused the polymer film to become cloudy due to the interaction with water vapor in the air. Liquid–liquid demixing occurred on top of the cast polymer through nucleation and growth, resulting in the formation cellular pores with interconnected structure. As a result, porous structure with symmetric membrane was obtained at higher exposure time.^30^

#### 3.2.3 Effect of air humidity

**Fig. 8:**
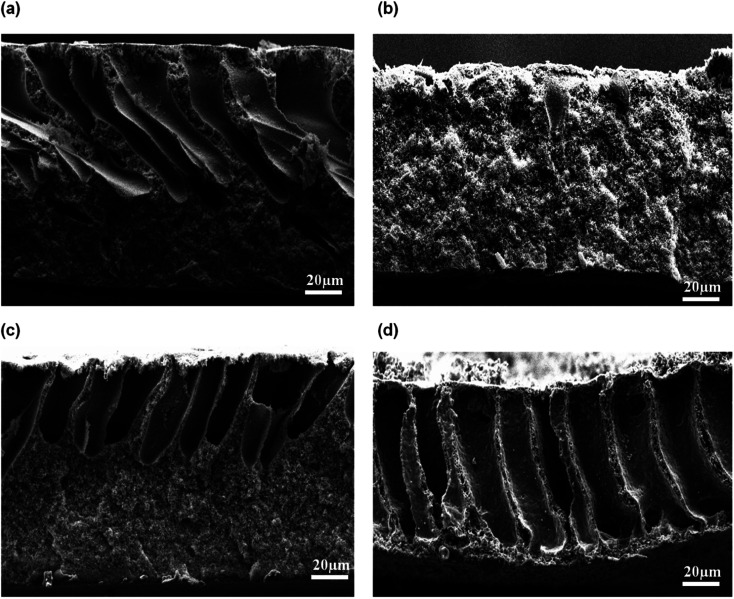
Cross-sectional of flat sheet membrane prepared at different air humidities: (a) 70%, (b) 80%, (c) 90%, and (d) 100%.

Fig. 8 exhibits the SEM micrograph of the membrane prepared at relative humidity between 70% and 100%. At low relative humidity of 70%, the long finger-like macrovoids were formed, originating from the top membrane surface and extended to more than half of the membrane's overall thickness. At 80% air humidity, the macrovoids had completely disappeared but formed a symmetric membrane with microporous cellular structure. Interestingly, when the humidity was further increased to 90%, the macrovoids reappeared but the size of the finger-like macrovoids were shorter compared to the membrane prepared at relative humidity of 70%. Membrane prepared at 100% relative humidity produced a large macrovoids from top to bottom part of membrane. Based on the physical observation during the casting process, the cast polymer film was optically clear and smooth prior to the immersion into coagulation bath at medium humidity of 70% and 80%. However, at high relative humidity of 90% and 100%, the cast film polymer immediately turned into cloudy film and the membrane surface became wrinkled after being immersed into the coagulation bath. Water intake is dominated over the solvent evaporation of the cast film at medium humidity. After some exposure time in the humid air, the cast film polymer was saturated with water vapor and the phase separation occurred before its immersion into the coagulation bath. At this condition, coarsening process that had occurred produced a bicontinuous structure with cellular micropores.^31^ Coarsening effect is the process of formation droplets of one phase dispersed in the matrix of a second phase. Fast exchange of the water vapor and solvent in the film polymer might have occurred at high relative humidity due to large existence of water vapor in air. Hence, it will form large macrovoids in the membrane.^31^

### 3.3 Membrane porosity

**Fig. 9:**
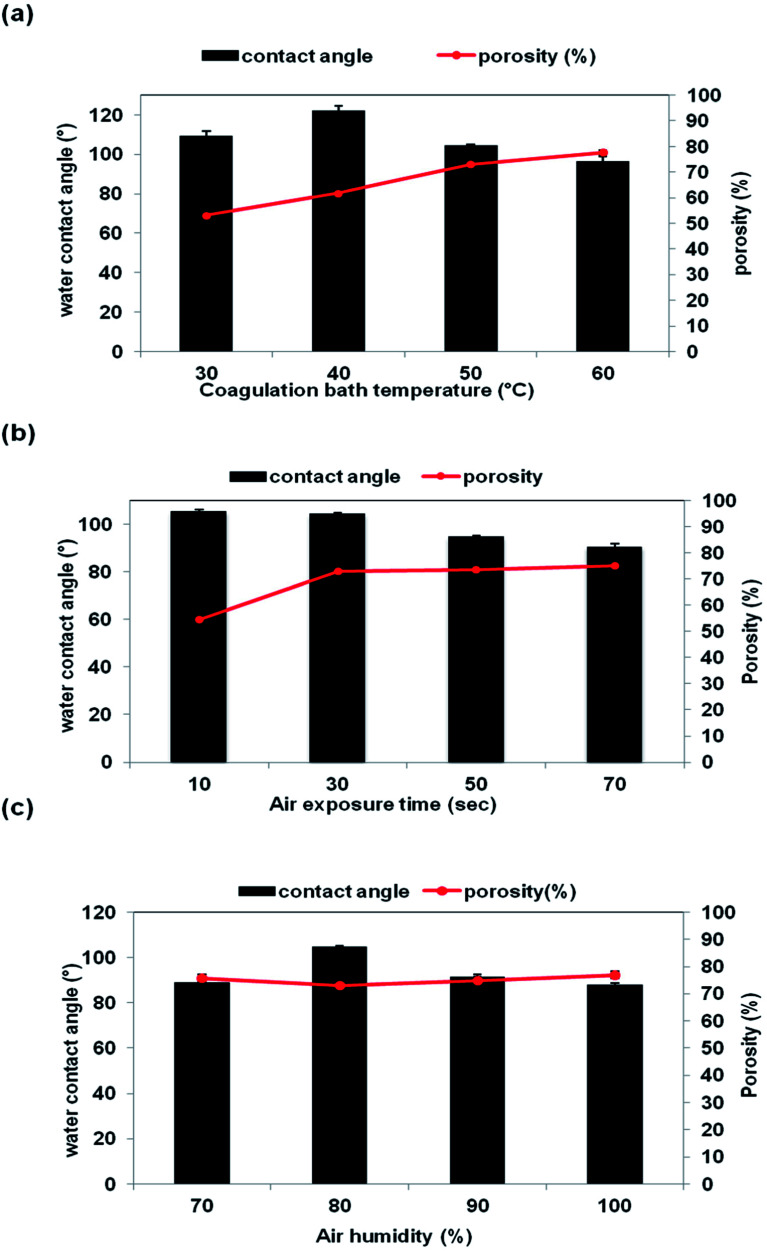
Porosity (%) and contact angle (°) of the membrane support prepared at different VIPS parameters: (a) CBT (b) air exposure time (c) air humidity.

The porosity of the membrane is influenced by several factors such as the number of pores, pore size, tortuosity, and polarity.^32^ Generally, large pore size indicates that the membrane contains a lot of empty spaces inside and around the pores, hence resulting in high membrane porosity.^33^ The effect of CBT, air exposure time and air humidity on the membrane porosity is shown in Fig. 9. The porosity of the membrane was increased along with the CBT and exposure time, as clearly seen in Fig. 9(a) and (b). Previously, based on the SEM images in Fig. 5 and 7, it shows that the interconnection between the cellular pores and number of pores increased with increasing CBT and air exposure time, thus leading to high porosity value. Beside, large macrovoids form from top to bottom membrane at high humidity also lead to increasing porosity value as shown in Fig. 8. This trend can be related to the porous morphology of resultant membrane. High CBT had delayed the demixing process and reduced the polymer precipitation rate. Thus, it had led to the formation of porous cellular structure.^29,34^ Meanwhile, increasing the air exposure time to more than 30 s had provided sufficient time for water absorption to cause phase separation in the entire film. At exposure time more than 30 s, the mass transfer was slow in exposure stage, with the water intake from air, PES had sufficient time to crystallize and produce porous membrane.^16^

The membrane porosity is not related linearly with the air humidity, as shown in Fig. 9(c). The membrane porosity initially increased from 70% air humidity up to 80%. At 90% air humidity, the porosity decreased, but then increased back to 100% air humidity. The changes of porosity value of the membrane prepared at different air humidities have correlated well with the membrane morphology shown and explained previously in Fig. 8. Large macrovoids appeared at 70% air humidity and disappeared at 80% but the number of interconnected micropores was increased. Then, macrovoids reappeared at air humidity from 90% to 100%.

### 3.4 Contact angle properties

Fig. 9(a)–(c) show the water contact angle value as a function of CBT, exposure time and air humidity, respectively. The contact angle of the membrane was decreased, which meant less hydrophobicity when the CBT exposure time and air humidity were increased. The contact angle was reduced from 122° to 97° as the CBT increased from 40 °C to 60 °C. When the air exposure time increased from 10 s to 70 s, the contact angle of the membrane reduced from 106° to 95°. The contact angle reduction can be related with the changes on the pores structure and porosity of the membrane. An increment of the CBT and exposure time increased the porosity of the membrane, as shown in Fig. 9(a) and (b). Porous membrane exhibited lower contact angle compared to a dense membrane.^27^ As for the air humidity, the contact angle value decreased from 105° to 88° when the air humidity increased from 70% to 100%. The drop in contact angle can be related to the existence of large macrovoids in the membrane structure, especially for the membrane prepared at 100% humidity.

### 3.5 SLM performance for acetic acid removal

#### 3.5.1 Effect of coagulation bath temperature

**Fig. 10:**
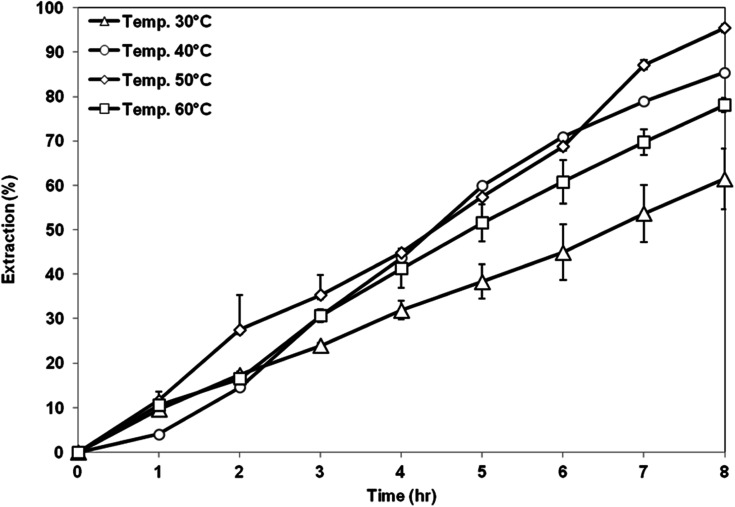
Extraction of AA using PES–graphene membrane support fabricated at different CBT.

Fig. 10 shows the extraction performance of AA from aqueous solution using membrane support prepared at different CBTs. The highest extraction of AA was obtained by using membrane support prepared at CBT of 50 °C with 95% of AA removal. Meanwhile, the lowest extraction value was shown by the membrane prepared at CBT of 30 °C. Based on the membrane characteristic, it shows that the membrane prepared at CBT 50 °C has a high porosity with bicontinuous cellular pores, symmetric structure and good hydrophobicity. Interconnected pores enhanced the flux and permeability of the membrane, thus enhancing the permeation of AA solute. In addition, symmetric membrane is more suitable in the SLM process because it has higher stability compared to that of asymmetric membrane. The force that exerted on both sides of the symmetric membranes is likely to be almost the same, thus, there is the possibility of improving the overall SLM process.^35^ Further increase of CBT to 60 °C had resulted in decreasing of the AA removal to 78%. Although the porosity of the membrane increased, which is good to accommodate more organic liquid membrane phase, its hydrophobicity was reduced. The liquid membrane became unstable and was unable to be retained within the membrane pores, thus, the solute was unable to be transferred through the membrane support efficiently.^12^

#### 3.5.2 Effect of air exposure time

**Fig. 11:**
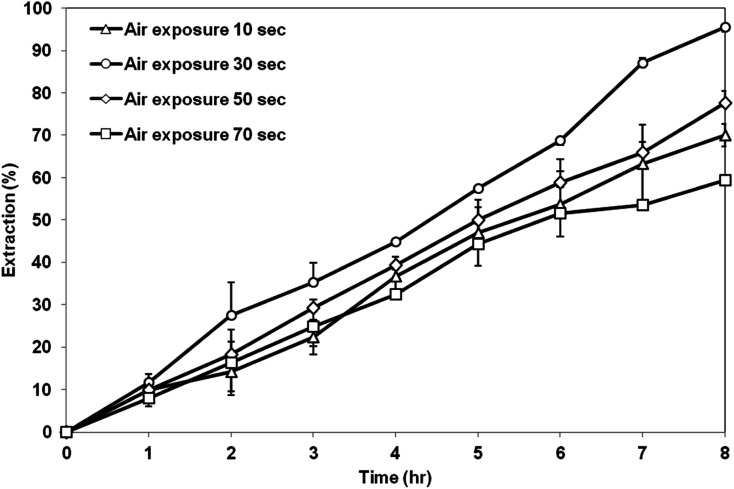
Extraction of AA using PES–graphene membrane support fabricated at different air exposure time.

Fig. 11 exhibits the extraction of AA using the membrane prepared at different air exposure time from 10 to 70 s. The highest AA extraction was achieved by the membrane support prepared at air exposure time of 30 s. Further increase in the air exposure time up to 70 s had decreased the extraction efficiency. Based on the SEM image shown previously, at low exposure time of 10 s, asymmetric membrane structure was formed, which is not preferable as a support for the SLM process. Air exposure time above 30 s produced symmetrical and micropores membranes structure. The porosity did not change too much as the exposure time increased but the contact angle value had decreased. Therefore, the extraction of AA by the membrane prepared at exposure time of 30 s was the highest due to the high contact angle value compared to the membrane prepared at exposure time of 50 s and 70 s. High contact angle value can minimize the leakage of the organic liquid membrane from the membrane support and retain its stability in the membrane pore.^36^

#### 3.5.3 Effect of air humidity

**Fig. 12:**
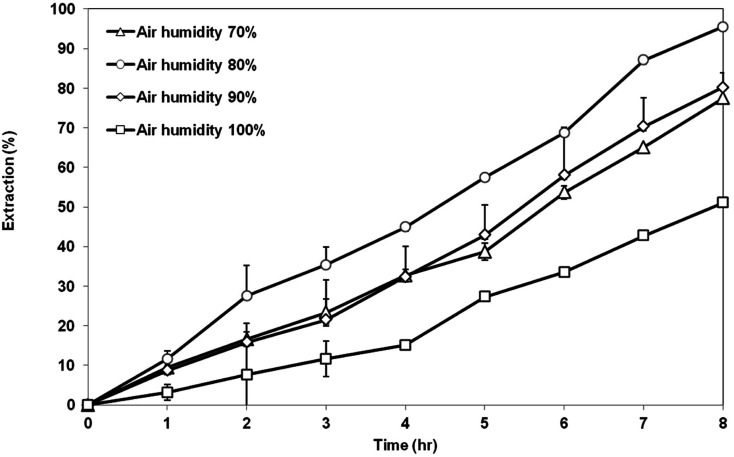
Extraction of AA using PES–graphene membrane support fabricated at different air humidities.

Fig. 12 exhibits the extraction of AA using the membrane prepared at different air humidity from 70 to 100%. Highest extraction of AA was achieved by the membrane support prepared at air humidity of 80%, which is around 95% AA removal. Membrane prepared at 80% air humidity had a suitable morphology and properties as the good support in the SLM process. The membrane has a symmetrical cellular micropores structure with balanced porosity and contact angle value. Lowest AA removal of 51% was shown by the membrane prepared at 100% air humidity. This is not surprising as seen previously in the SEM image that showed that the structure of the membrane consisted of a very large go-through macrovoids. The existence of large macrovoids can weaken the capillary force that is responsible for retaining the organic liquid membrane inside the pores of the membrane support. As a result, the liquid membrane can be easily washed out and eventually decrease the extraction of the AA. Membrane prepared at air humidity of 70% and 90% showed an asymmetric structure with low porosity. Therefore, the extraction value for both membranes is less than that of membrane prepared at 80% humidity, but still better than the membrane prepared at 100% humidity.

### 3.6 Membrane stability in SLM process

**Fig. 13:**
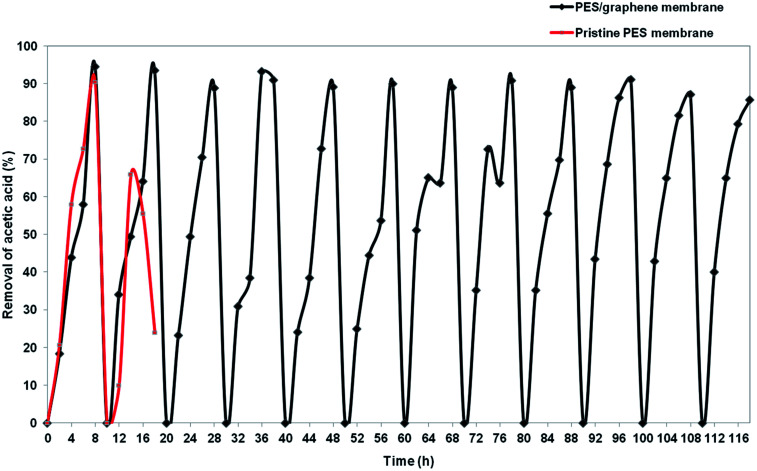
Stability of pristine PES membrane and hybrid PES–graphene membrane as a SLM support.

Insufficient stability of the membrane support is one of major problem that needs to be solved before applying SLM at industrial scale. Hybrid PES–graphene prepared at CBT 40 °C, 30 s of exposure time and 80% of air humidity was used in stability study to compare with pristine PES membrane. Fig. 13 exhibits the stability of pristine PES membrane and hybrid PES–graphene membrane during SLM process. The stability of the pristine PES membrane diminished after two SLM cycles (16 h). The removal percentage of AA drop from 90.4% to 56%. The breakage of pristine PES membrane had occurred which caused the fluctuation of the feed and strip phase solution. Pristine PES membrane had showed insufficient tensile strength and low chemical resistance. Corrosive chemicals can erode the surface of the membrane during SLM until breakage of the membrane support was occurred.^37^ As a result, the liquid membrane forced out from the membrane phase and progressive wetting of the membrane pores by aqueous solution take placed.^12^

For hybrid PES–graphene membrane, it was observed that the membrane support remains stable during twelve SLM cycles (116 h). 94% of AA removal was achieved during first two SLM cycles and then the removal percentage was maintained at average value of 90%. Hybrid PES–graphene membrane had showed high stability for continuous run without required re-impregnation of the organic liquid membrane into the membrane support. The addition of graphene significantly improved the stability of SLM due to enhancement of tensile strength and hydrophobicity of the membrane. High hydrophobicity and high mechanical strength of the membrane support can extend the lifetime of SLM process by dramatically reduce the loss of liquid membrane and inhibit the water flooding in the membrane surface.^38^

## 4 Conclusion

Microporous hybrid PES–graphene membrane membranes have been successfully prepared by the VIPS method at different coagulation bath temperatures, air exposure times and air humidities. The membrane prepared at a coagulation bath temperature of 50 °C, air exposure time of 30 s and air humidity of 80% exhibited a symmetric structure with microporous cellular pores, high hydrophobicity, high porosity and high mechanical strength. Due to this suitable morphology and properties of this membrane, almost 95% of AA was successfully removed from an aqueous solution using the SLM process. The hybrid PES–graphene membrane remains stable for more than 116 h compared to the pristine PES membrane that only showed 16 h stability. Hence, the hybrid membrane has high potential to be applied in real industrial application.

## Conflicts of interest

There are no conflicts to declare.

## Supplementary Material
